# Chronic Ethanol Consumption Impairs the Tactile-Evoked Long-Term Depression at Cerebellar Molecular Layer Interneuron-Purkinje Cell Synapses *in vivo* in Mice

**DOI:** 10.3389/fncel.2018.00521

**Published:** 2019-01-14

**Authors:** Da-Yong Li, Yan-Hua Bing, Chun-Ping Chu, Xun Cui, Song-Biao Cui, De-Lai Qiu, Li-Da Su

**Affiliations:** ^1^Key Laboratory of Cellular Function and Pharmacology of Jilin Province, Yanbian University, Yanji, China; ^2^Department of Physiology and Pathophysiology, College of Medicine, Yanbian University, Yanji, China; ^3^College of Basic Courses, Zhejiang Shuren University, Hangzhou, China; ^4^Department of Neurology, Affiliated Hospital of Yanbian University, Yanji, China; ^5^Neuroscience Care Unit, Second Affiliated Hospital of Zhe-Jiang University School of Medicine, Hangzhou, China

**Keywords:** ethanol, cerebellar purkinje cell, molecular layer interneuron, sensory stimulation, plasticity, nitric oxide, *in vivo* cell-attached recording

## Abstract

The cerebellum is sensitive to ethanol (EtOH) consumption. Chronic EtOH consumption impairs motor learning by modulating the cerebellar circuitry synaptic transmission and long-term plasticity. Under *in vitro* conditions, acute EtOH inhibits both parallel fiber (PF) and climbing fiber (CF) long-term depression (LTD). However, thus far it has not been investigated how chronic EtOH consumption affects sensory stimulation-evoked LTD at the molecular layer interneurons (MLIs) to the Purkinje cell (PC) synapses (MLI-PC LTD) in the cerebellar cortex of living animals. In this study, we investigated the effect of chronic EtOH consumption on facial stimulation-evoked MLI-PC LTD, using an electrophysiological technique as well as pharmacological methods, in urethane-anesthetized mice. Our results showed that facial stimulation induced MLI–PC LTD in the control mice, but it could not be induced in mice with chronic EtOH consumption (0.8 g/kg; 28 days). Blocking the cannabinoid type 1 (CB1) receptor activity with AM-251, prevented MLI-PC LTD in the control mice, but revealed a nitric oxide (NO)-dependent long-term potentiation (LTP) of MLI–PC synaptic transmission (MLI-PC LTP) in the EtOH consumption mice. Notably, with the application of a NO donor, S-nitroso-N-Acetyl-D, L-penicillamine (SNAP) alone prevented the induction of MLI–PC LTD, but a mixture of SNAP and AM-251 revealed an MLI-PC LTP in control mice. In contrast, inhibiting NO synthase (NOS) revealed the facial stimulation-induced MLI-PC LTD in EtOH consumption mice. These results indicate that long-term EtOH consumption can impair the sensory stimulation-induced MLI–PC LTD *via* the activation of a NO signaling pathway in the cerebellar cortex *in vivo* in mice. Our results suggest that the chronic EtOH exposure causes a deficit in the cerebellar motor learning function and may be involved in the impaired MLI–PC GABAergic synaptic plasticity.

## Introduction

The cerebellum is an important organ controlling motor coordination, planning and fine regulating voluntary movement, and is also critical for cognitive functions, such as thought, behavior and emotion. The cerebellar cortex is composed of a molecular layer, a Purkinje cell (PC) layer and a granule layer, and these three layers mainly include PC, molecular layer interneurons (MLIs), granule cells (GCs) and Golgi cells (Palay and Chan-Palay, 1974). The PC is the focus of computation in the cerebellar cortex, receiving convergent projections from all other cortical neurons and providing the sole output from the cerebellar cortex to the deep cerebellar nuclei (Palay and Chan-Palay, 1974). The cerebellum is a target of the actions of ethanol (EtOH; Luo, 2012). EtOH consumption causes alterations of motor coordination, balance, behavior, speech, and certain cognitive functions. which are considered to be caused partly by impairment of cerebellar circuit functions and the modulation of synaptic transmissions (Schmahman and Sherman, 1997; Mameli et al., 2008). EtOH is known to enhance GABA–mediated synaptic transmission and to inhibit glutamatergic synaptic transmission (Lovinger, 1997; Woodward, 1999). It has been assumed that some of the behavioral actions of EtOH are mediated by enhancing the inhibitory action of GABA (Martz et al., 1983; Criswell and Breese, 2005; Weiner and Valenzuela, 2006; Botta et al., 2010). For instance, EtOH increases the frequency of miniature and spontaneous inhibitory postsynaptic currents at PCs and MLIs in rat cerebellar slices, *via* increasing the GABA release (Mameli et al., 2008; Hirono et al., 2009; Wadleigh and Valenzuela, 2012), and modulating facial stimulation-evoked GABAergic responses in the mouse cerebellar cortical molecular layer (Cui et al., 2014).

In cerebellar cortical circuits, long-term synaptic plasticity could be induced at the parallel fiber-PC (PF-PC), the PF-MLIs, mossy fiber-GC (MF-GC) and MLI-PC synapses under *in vitro* conditions, which has been proposed to provide a cellular mechanism for motor learning (Grasselli and Hansel, 2014). Both long-term potentiation (LTP) and long-term depression (LTD) of the PF-PC synapses have been demonstrated previously (Ito, 1989; D’Angelo et al., 2016; Hoxha et al., 2016). PF-PC LTD is the earliest characterized form of synaptic plasticity in the cerebellar cortex (Ito, 1989), while PF-PC LTP is latterly found and expressed both pre- and post-synoptically (Qiu and Knöpfel, 2007; Anggono and Huganir, 2012). Long-term synaptic plasticity has been induced by postsynaptic depolarization at MLI-PC synapses *via* activation of the cannabinoid type I receptor (CB1) and the N-methyl-D-aspartate (NMDA) receptor in cerebellar slices. The MLI-PC synaptic plasticity induced by excitatory inputs, express hetero-synoptically (Hirano, 2013; Hirano and Kawaguchi, 2014). However, there was no similar MLI-PC synaptic plasticity observed under *in vitro* conditions. The reason may be related to the difficulty of recording the electrical stimulation-evoked MLI-PC inhibitory postsynaptic currents in cerebellar slices. Therefore, MLI-PC synaptic plasticity induced by the MLIs inhibitory input is less known under *in vitro* conditions.

It has been reported that EtOH impaired long-term synaptic plasticity in the hippocampus (Zorumski et al., 2014) and the cerebellum (Chandler et al., 1998; Belmeguenai et al., 2008; Su et al., 2010; He et al., 2013). Administration of EtOH during standard stimulation inhibited both LTP and LTD in the CA1 region of a rat hippocampus (Izumi et al., 2005, 2007; Tokuda et al., 2011). The effects of EtOH on the hippocampal LTD were diminished by the blockade of NMDA receptors, while the effects of EtOH on the hippocampal LTP were involved in both NMDA receptors and GABAergic transmission (Izumi et al., 2007). Furthermore, chronic intermittent consumption of EtOH disrupts the NMDA receptor-associated post-synaptic proteins and specifically regulates group I mGlu receptor-dependent LTD in the mouse hippocampus (Wills et al., 2017). Moreover, chronic EtOH consumption induces a reduced-performance in a spatial recognition task in normal animals, but it attenuates spatial memory deficits and increases group I mGlu receptor expression in the rat hippocampus (Van Waes et al., 2009). In the cerebellum, it has been described that acute EtOH selectively blocked PF–LTD induction, whereas it did not change the amplitude of excitatory postsynaptic currents at the PF synapse *in vitro* in mice (Belmeguenai et al., 2008). Application of EtOH at a concentration of 50 mM inhibited LTD at the climbing fiber (CF) synapses onto PCs *via* inhibition of the NMDA receptors and the group I mGlu receptors (Carta et al., 2006; Belmeguenai et al., 2008; Su et al., 2010; He et al., 2013). However, it has been suggested that acute EtOH also impaired the induction of LTP, possibly through several other mechanisms that include the inhibition of the group I mGlu receptor-mediated potentiation of the NMDA receptor function and of the evoked dopamine release in the mouse nucleus accumbens (Mishra et al., 2012). Chronic EtOH exposure significantly reduced simple and complex spike frequencies of PCs, resulting in a depression of cerebellar motor coordination and ataxia in mice (Servais et al., 2005).

In addition, systemic administrations of EtOH resulted in a dose-dependent increase in nitric oxide (NO) levels, which was attenuated by the NO synthase (NOS) inhibitor, indicating that systemic administration of EtOH increased brain NO levels (Finnerty et al., 2015). Acute treatment with EtOH increased NOS activity and NO production in brain memory related regions, such as the prefrontal cortex, amygdala and the hippocampus in adult mice (Yu et al., 2013). Recently, it has been demonstrated that EtOH caused an increase in the level of glutamate, NO, and GABA in the rostral ventrolateral medulla during the hypotensive responses, suggesting that EtOH enhanced glutamatergic NMDA receptors /NO/GABA pathways in the rostral ventrolateral medulla and may participate in the hypotensive effects induced by acute administration of EtOH (Lo et al., 2018). NO also has been involved in cerebellar cortical PF-PC presynaptic LTP under *in vitro* (Qiu and Knöpfel, 2007) and *in vivo* conditions (Chu et al., 2014).

We previously found that facial stimulation evoked strong MLI-PC synaptic transmission *in vivo* in mice (Chu et al., 2011a,b, 2012). The facial stimulation evoked spike firing in MLIs, resulting in an inhibitory response in cerebellar PCs *via* a MF-GC pathway, but did not evoke complex spikes in PCs *via* CFs. The facial stimulation evoked a simple spike firing in PCs only in the presence of the GABA_A_ receptors antagonist (Chu et al., 2012). Furthermore, we found that facial stimulation induced LTD at MLI-PC synapses (MLI-PC LTD), accompanied with a decrease in the stimulation-evoked pause of spike firing in PCs *via* activation of NMDA receptors in the mouse cerebellar cortex, suggesting that sensory stimulation evoked MLI-PC GABAergic synaptic plasticity may play a critical role in motor learning of living animals (Bing et al., 2015). Moreover, acute application of EtOH inhibits the facial stimulation evoked MLI-PC synaptic transmission (Cui et al., 2014), and significantly depresses the MLI-PC synaptic transmission by activating presynaptic cannabinoid receptors *via* the protein kinase signaling pathway, suggesting that EtOH modulates GABA release from MLIs onto PCs (Wu et al., 2016). Our previous studies suggest that the cerebellar MLI-PC synapse is a target of EtOH, and EtOH consumption may impair the MLI-PC synaptic plasticity.

Altogether, EtOH exposure impaired long-term synaptic plasticity at PF-PC and CF-PC synapses in the cerebellar cortex, have been demonstrated under *in vitro* conditions. However, the effects of chronic EtOH exposure on the sensory stimulation-evoked MLI-PC LTD in the cerebellar cortex of living animals, are currently unknown. Therefore, we investigated the effects of chronic EtOH consumption on facial stimulation-evoked MLI-PC LTD, using electrophysiological techniques and pharmacological methods in urethane-anesthetized mice.

## Materials and Methods

### Animals

A total of 56 (5-week-old) HA/ICR mice were used in this study. The mice were divided into the EtOH consumption group (29 mice) and the control group (27 mice). The experimental procedures were approved by the Animal Care and Use Committee of the Yanbian University and were in accordance with the animal welfare guidelines of the U.S. National Institutes of Health. The permit number is SYXK (Ji) 2011-006. All animals were housed under a 12-h light: 12-h dark cycle with free access to food and water in a colony room under constant temperature (23 ± 1°C) and humidity (50 ± 5%). Mice in the EtOH consumption group were intraperitoneally (i.p.) injected with EtOH (0.8 g/kg; 15% in saline), while mice in the control group were i.p. injected with the same volume of saline. The EtOH (95%) was diluted in saline for a final concentration of 15%. The electrophysiological recordings were performed after the i.p. injection of EtOH, for 28 days.

### Anesthesia and Surgical Procedures

The anesthetic and surgical procedures have been described previously (Chu et al., 2011a,b). The mice were anesthetized with urethane (1.3 g/kg body weight, i.p.), and were tracheotomized to avoid respiratory obstruction. On a custom-made stereotaxic frame, soft tissue was retracted to gain access to the dorsal portion of the occipital bone. A watertight chamber was created and a 1–1.5 mm craniotomy was drilled to expose the cerebellar surface corresponding to Crus II. The brain surface was constantly superfused with oxygenated artificial cerebrospinal fluid (ACSF: 125 mM NaCl, 3 mM KCl, 1 mM MgSO_4_, 2 mM CaCl_2_, 1 mM NaH_2_PO_4_, 25 mM NaHCO_3_, and 10 mM D-glucose) with a peristaltic pump (Gilson Minipulse 3; Villiers, Le Bel, France) at 0.4 ml/min. Rectal temperature was monitored and maintained at 37.0 ± 0.2°C using body temperature equipment.

### Cell-Attached Recording and Facial Stimulation

Cell-attached recordings from cerebellar PCs were performed with an Axopatch-200B amplifier (Molecular Devices, Foster City, CA, USA). The signals of PC cell-attached recordings were acquired through a Digidata 1440 series analog-to-digital interface on a personal computer using Clampex 10.3 software (Molecular Devices). Patch pipettes were made with a puller (PB-10; Narishige, Tokyo, Japan) from thick-wall borosilicate glass (GD-1.5; Narishige). Recording electrodes were filled with ACSF, with a resistances of 3–5 MΩ. The cell-attached recordings from PCs were performed at depths of 200–300 μm under the pia mater membrane, and were identified by regular spontaneous simple spikes (SS) accompanied with irregular complex spikes.

**Figure 1:**
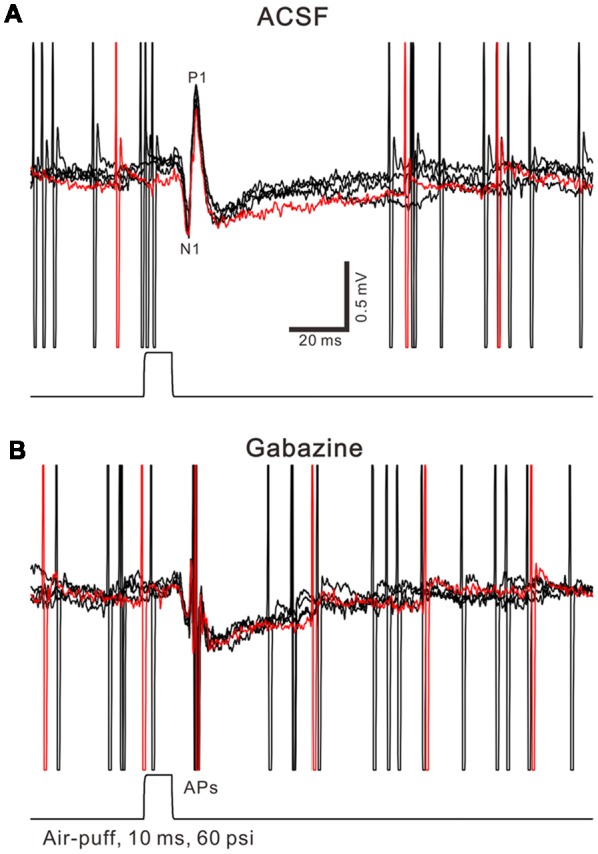
Property of facial stimulation-evoked responses in cerebellar Purkinje cells (PCs). **(A)** Under cell-attached recording conditions (current-clamp mode; *I* = 0 pA), superposition of five sweeps shows a cerebellar PC in response to the tactile stimulation (bar, 10 ms) in artificialcerebrospinal fluid (ACSF). **(B)** The superposition of five sweeps shows the PC in response to the tactile stimulation (bar, 10 ms) in the presence of gabazine (50 μM). APs denote action potentials.

Facial stimulation was performed by air-puff (10 ms, 60 psi) of the ipsilateral whisker pad through a 12-gauge stainless steel tube connected with a pressurized injection system (Picospritzer^®^ III; Parker Hannifin Co., Pine Brook, NJ, USA). The air-puff stimuli were controlled by a personal computer, and were synchronized with the electrophysiological recordings and delivered at 0.05 Hz *via* a Master 8 controller (A.M.P.I., Jerusalem, Israel) and Clampex 10.3 software. The facial stimulation-evoked MLI–PC synaptic response has been demonstrated in our previous studies (Chu et al., 2011b; Bing et al., 2015), the response is expressed as a sequence of negative components (N1) followed by a positive component (P1) accompanied with a pause of SS firing (Figure 1A). N1 is identified as parallel fiber volley, while P1 is identified as MLI-PC synaptic transmission evoked by the facial stimulation through the MF-GC pathway (Chu et al., 2011b). For induction of MLI-PC synaptic plasticity, air-puff stimulation (10 ms, 60 psi; 240 pulses, 1 Hz) was delivered 10 min after the recording became stable.

### Chemicals

The reagents, which included N-(piperidin-1-yl)-5-(4-iodophenyl)-1-(2,4-di-chlorophenyl)-4-methyl-1H-pyrazole-3-carboxamide (AM251), CB1 receptors antagonist; N^G^-Nitro-L-arginine (L-NNA), NOS inhibitor; S-nitroso-N-Acetyl-D, L-penicillamine (SNAP), and NO donor. All chemicals were purchased from Sigma-Aldrich (Shanghai, China). For experiments with L-NNA and SNAP, the cerebellar surface was perfused with 200 μM L-NNA and 100 μM SNAP for 1 h before recordings were started, respectively. The drugs were dissolved in ACSF, and applied directly onto the cerebellar surface by a peristaltic pump (0.5 ml/min).

### Data Analysis

The electrophysiological data were analyzed using Clampfit 10.3 software (Molecular Devices, Foster City, CA, USA). The amplitude of P1 before and after 1 Hz facial stimulation was normalized by the mean value of the baseline. Values are expressed as the mean ± SEM. One-way analyses of variance (ANOVA; *post hoc* multiple comparison) and two-way ANOVA (SPSS Software) was used to determine the level of statistical significance among the groups of data. *P*-values below 0.05 were considered statistically significant.

## Results

### Effect of Chronic EtOH Consumption on Facial Stimulation Induced MLI-PC LTD in the Cerebellar Cortex

**Figure 2:**
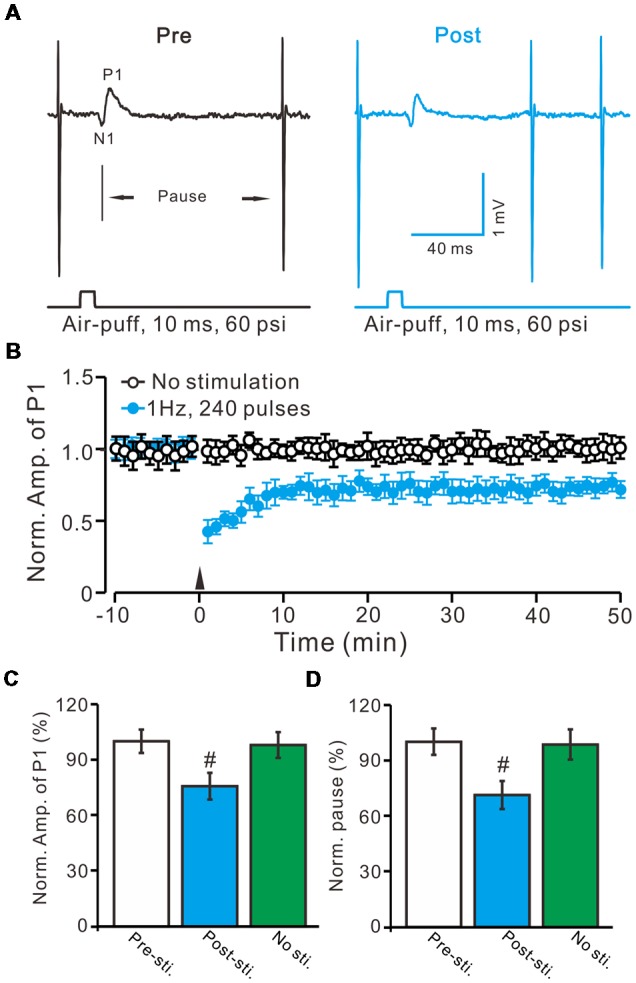
Facial stimulation at 1 Hz induces molecular layer interneuron-PC long-term depression (MLI-PC) LTD in the cerebellar cortex of control mice. **(A)** Representative cell-attached recording traces, showing air-puff stimulation (10 ms, 60 psi)-evoked responses in a cerebellar PC before (Pre) and after (post) delivering 1 Hz (240 pulses) stimulation. **(B)** Summary of the normalized positive component (P1) amplitude under control conditions (No stimulation, open circle; *n* = 8 mice) and delivery of 1 Hz facial stimulation (arrow head; filled circles; *n* = 8 mice). **(C)** Bar graph (*n* = 8 mice) showing the normalized amplitude of P1 before (Pre sti.) and after (Post sti.) delivery of 1 Hz stimulation and no stimulation (No sti). **(D)** Summary of data (*n* = 8 mice) showing the normalized pause of a simple spike firing before (Pre sti.) and after (Post sti.) delivery of 1 Hz stimulation and no stimulation (No sti.). Note that facial stimulation at 1 Hz induced MLI-PC LTD in mouse cerebellar cortex. Data points are mean ± SEM. ^#^*P* < 0.05 vs. baseline (Pre-sti.).

Air-puff stimulation on the ipsilateral whisker pad (10 ms; 60 psi) evoked an inhibitory component (P1) followed by a pause of SS firing (Figure 1A). The mean latency of N1 was 16.4 ± 0.22 ms, while the mean latency of P1 was 19.1 ± 0.34 ms (*n* = 27 control mice). Application of the GABAA receptors antagonist, Gabazine (50 μM) abolished P1 and revealed the facial stimulation-evoked action potentials (Figure 1B). According to our previous studies (Chu et al., 2011b, 2012), P1 is identified as MLI-PC GABAergic synaptic transmission onto cerebellar PCs. Consistent with our previous study (Bing et al., 2015), facial stimulation at 1 Hz (240 pulses) produced an MLI-PC LTD, which was expressed as a decrease in P1 amplitude and a pause of SS for over 50 min in control mice (Figures 2A,B). The normalized amplitude of P1 was decreased to 75.5 ± 7.2% of the baseline for 40–50 min after 1 Hz facial stimulation (*P* < 0.05, *n* = 8, Figure 2C). The normalized value of the SS pause, at 40–50 min after 1 Hz facial stimulation, was decreased to 71.2 ± 7.6% of the baseline (100.0 ± 7.1%; *P* < 0.05, *n* = 7, Figure 2D). These results indicate that 1 Hz facial stimulation induces MLI-PC LTD in the cerebellar cortex of control mice.

**Figure 3:**
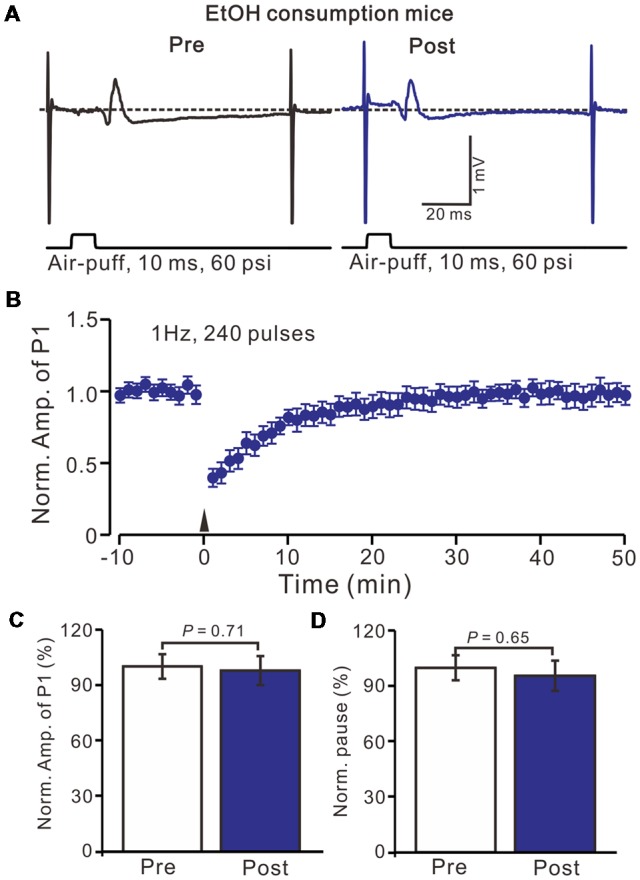
Facial stimulation at 1 Hz failed to induce MLI-PC LTD in ethanol (EtOH) consumption mice.** (A)** Representative traces showing air-puff stimulation (10 ms, 60 psi)-evoked responses in a PC from an EtOH consumption mouse before (left) and after delivering 1 Hz stimulation. **(B)** Summary of the normalized P1 amplitude under delivery of 1 Hz facial stimulation (arrow head; *n* = 8 mice). **(C)** Bar graph (*n* = 8 mice) showing the normalized amplitude of P1 before (Pre) and after (Post) delivery of 1 Hz stimulation.** (D)** Summary of data (*n* = 8 mice) showing the normalized pause of a simple spike firing before (Pre) and after (Post) delivery of 1 Hz stimulation. Note that air-puff stimulation at 1 Hz failed to induce MLI-PC LTD in EtOH consumption mice. Data points are mean ± SEM.

Acute EtOH impairs long-term synaptic plasticity at PF-PC synapses (Belmeguenai et al., 2008; Su et al., 2010) and CF-PC synapses (He et al., 2013) have been described in rodent cerebellar slices, suggesting that long-term EtOH consumption might impair MLI-PC synaptic plasticity *in vivo* in mice. EtOH consumption for 28 days with facial stimulation at 1 Hz (240 pulses) failed to produce an MLI-PC LTD in the cerebellar cortex of mice (Figures 3A,B). The normalized amplitude of P1 was 97.6 ± 7.8% of the baseline for 40–50 min after 1 Hz facial stimulation (*P* > 0.05, *n* = 7, Figure 3C). The normalized value of the SS pause, at 40–50 min after 1 Hz facial stimulation was 95.2 ± 8.1% of the baseline (100.0 ± 6.7%; *P* > 0.05, *n* = 7, Figure 3D). These results indicate that 1 Hz facial stimulation fails to induce MLI-PC LTD in chronic EtOH consumption mice, suggesting that long-term consumption of EtOH impairs MLI-PC LTD *in vivo* in mice.

### MLI-PC LTD Is Dependent on CB1 Receptors Activity

**Figure 4:**
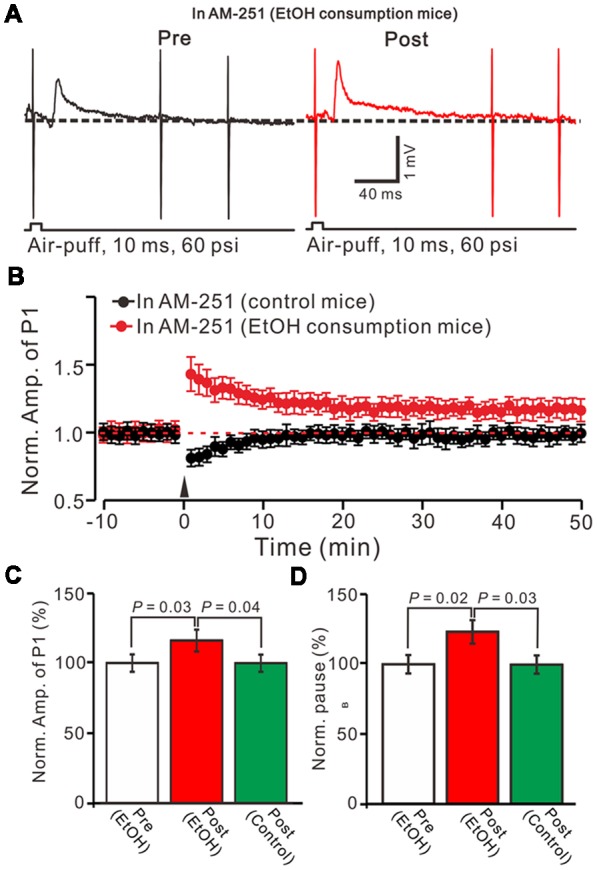
Blocking cannabinoid type 1 (CB1) receptors activity, facial stimulation failed to induce MLI-PC LTD in control mice, but induced MLI-PC long-term potentiation (LTP) in EtOH consumption mice. **(A)** Representative cell-attached recording traces showing air-puff stimulation (10 ms, 60 psi)-evoked responses in a cerebellar PC from an EtOH consumption mouse before (left) and after delivering 1 Hz (240 pulses) stimulation in the presence of a CB1 antagonist, AM251 (5 μM). **(B)** Summary of the normalized P1 amplitude after delivery of 1 Hz facial stimulation (arrow head) in control mice (black; *n* = 7 mice) and EtOH consumption mice (Red; *n* = 7). **(C)** Bar graph (*n* = 7 mice) showing the normalized amplitude of P1 before (Pre EtOH) and after (Post, EtOH; Post control) delivery of 1 Hz stimulation in EtOH consumption and control mice.** (D)** Summary of data (*n* = 7 mice) showing the normalized pause of a simple spike firing before and after delivery of 1 Hz stimulation in EtOH consumption and control mice. Note that blocking by CB1 receptor activity, facial stimulation failed to induce MLI-PC LTD in control mice, but induced MLI-PC LTP in EtOH consumption mice.

In the cerebellar cortex, trains of PF stimulation can induce endocannabinoid (eCB) generation and release from PCs and MLIs through the activation of group I mGuR and NMDA receptors (Brown et al., 2003; Beierlein and Regehr, 2006; Soler-Llavina and Sabatini, 2006), which are considered to be involved in PF–PC presynaptic plasticity (Qiu and Knöpfel, 2009; Chu et al., 2014) and MLI–PC LTD (Bing et al., 2015). Therefore, we examined the effect of the CB1 receptor antagonist on the induction of 1 Hz facial stimulation-induced MLI-PC LTD in both the control mice and EtOH consumption mice. Blocking CB1 receptor activity with AM251, completely prevented the 1 Hz facial stimulation induced MLI–PC LTD in control mice. However, by blocking the CB1 receptors activity, facial stimulation induced a LTP of MLI-PC synaptic transmission (MLI-PC LTP) in EtOH consumption mice (Figures 4A,B). In the presence of AM251, the mean amplitude of P1 was 116.1 ± 7.8% (*n* = 7) of the baseline (100 ± 6.3%; Figures 3A,C; *P* = 0.03; *n* = 7) at 40–50 min after the trains of 1 Hz facial stimulation was delivered in the EtOH consumption mice. The mean normalized amplitude of P1 was significantly higher than that in the control mice (98.6 ± 6.1%; *n* = 8; Figures 4A,C; *P* = 0.04). Moreover, the mean pause of SS firing was 123.3 ± 8.4% (*n* = 7) of the baseline (100 ± 6.3%; Figures 4A,D; *P* = 0.02) at 40–50 min after the trains of 1 Hz facial stimulation was delivered in the EtOH consumption mice, which was significantly longer than that in the control mice (99.6 ± 6.5%; *n* = 8; Figures 4A,D; *P* = 0.03). These results indicate that by blocking the CB1 receptors activity, facial stimulation fails to induce MLI-PC LTD in control mice, but induces MLI-PC LTP in EtOH consumption mice.

### NO Involves in the Facial Stimulation Induced MLI-PC Plasticity in EtOH Consumption Mice

**Figure 5:**
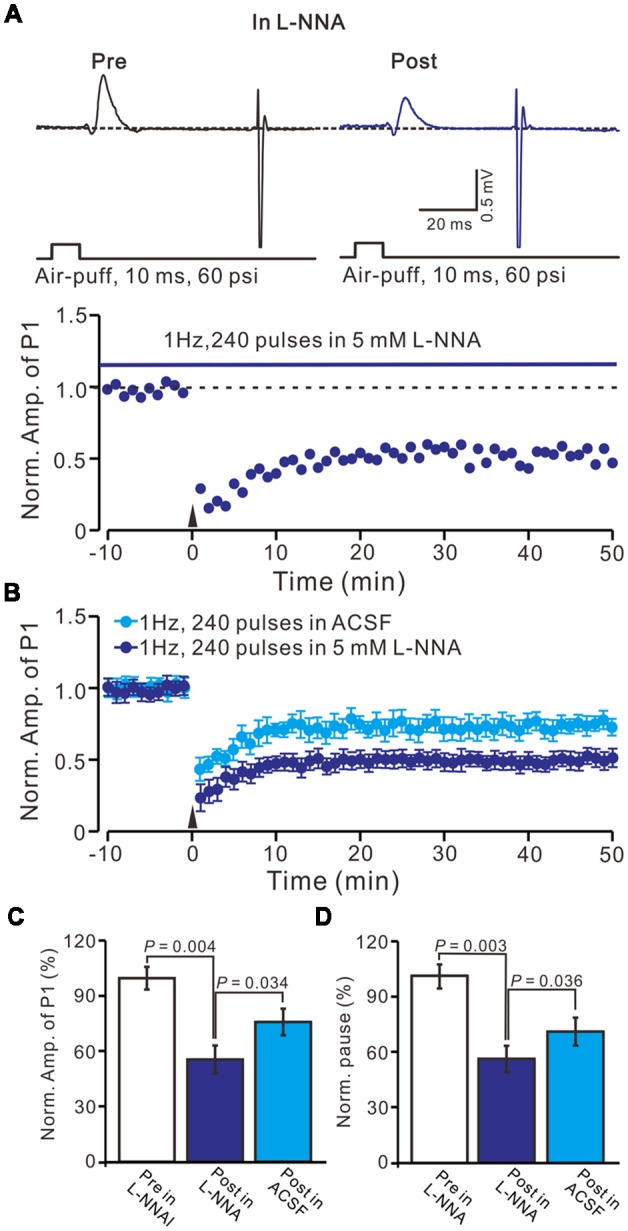
Inhibition of nitric oxide synthase (NOS), facial stimulation induced MLI-PC LTD in EtOH consumption mice. **(A)** In the presence of the NOS inhibitor, representative traces showing air-puff stimulation (10 ms, 60 psi) evoked responses in a cerebellar PC from an EtOH consumption mouse before (Pre) and after (Post) delivering 1 Hz (240 pulses) stimulation. **(B)** Summary of the normalized P1 amplitude after delivery of 1 Hz facial stimulation (arrow head) in treatment with ACSF (blue) and the NOS inhibitor (purple; *n* = 7 mice). **(C)** Bar graph (*n* = 7 mice) showing the normalized amplitude of P1 before (Pre) and after (Post) delivery of 1 Hz stimulation. **(D)** Summary of data (*n* = 7 mice) showing the normalized pause of a simple spike firing before (Pre) and after (Post) delivery of 1 Hz stimulation. Note that at the inhibition of NOS, facial stimulation induced MLI-PC LTD, in EtOH consumption mice.

We previously demonstrated that 4 Hz PF stimulation induced-PF-PC presynaptic LTP required activation of NOS under *in vitro* (Qiu and Knöpfel, 2007) and *in vivo* conditions (Chu et al., 2014), suggesting that NOS might be involved in the facial stimulation induced MLI-PC LTD under *in vivo* conditions. We then examined 1 Hz facial stimulation induced MLI-PC plasticity, in the presence of an NOS inhibitor, L-NNA (100 μM), in EtOH consumption mice. After the perfusion of L-NNA for 1 h, the 1 Hz facial stimulation induced MLI–PC LTD in EtOH consumption mice (Figures 5A,B). The mean amplitude of P1 was 76.8 ± 7.2% (*n* = 7) of the baseline (100 ± 6.2%; Figure 5C; *P* = 0.032; *n* = 7) at 40–50 min after the trains of 1 Hz facial stimulation were delivered in the EtOH consumption mice. The mean pause of SS firing was 77.2 ± 7.1% (*n* = 7) of the baseline (100 ± 5.6%; Figures 5A,D; *P* = 0.036) at 40–50 min after the trains of 1 Hz facial stimulation were delivered in the EtOH consumption mice. These results indicate that facial stimulation induces MLI-PC LTD in EtOH consumption mice when NOS is inhibited.

**Figure 6:**
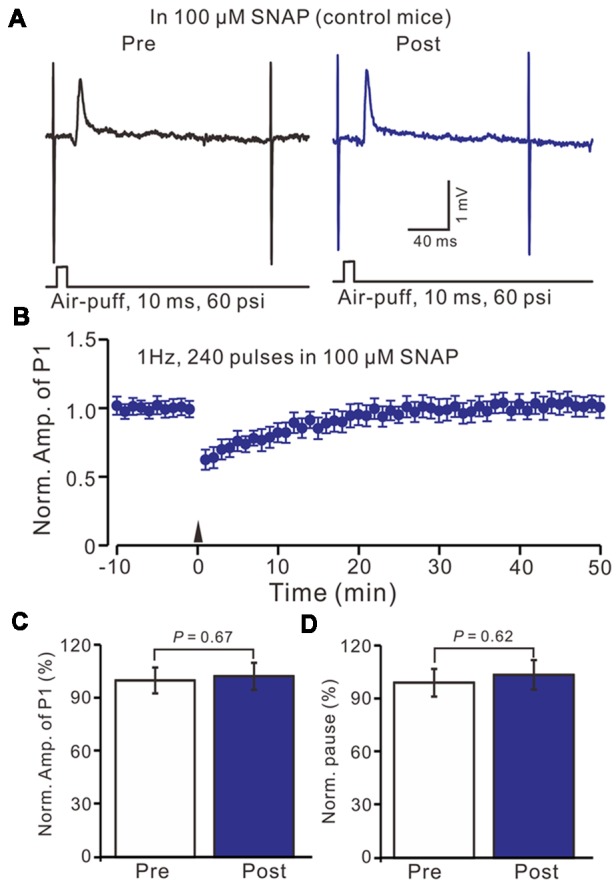
In the presence of NO donor, S-nitroso-N-Acetyl-D, L-penicillamine (SNAP; 100 μM), facial stimulation failed to induce MLI-PC LTD in control mice. **(A)** In the presence of the NO donor, representative traces showing air-puff stimulation (10 ms, 60 psi) evoked responses in a cerebellar PC from a control mouse before and after delivering 1 Hz stimulation. **(B)** Summary of the normalized P1 amplitude after delivery of 1 Hz facial stimulation (arrow head) in the presence of the NO donor in control mice (*n* = 7 mice). **(C)** Bar graph (*n* = 7 mice) showing the normalized amplitude of P1 before (Pre) and after (Post) delivery of 1 Hz stimulation. **(D)** Summary of data (*n* = 7 mice) showing the normalized pause of a simple spike firing before (Pre) and after (Post) delivery of 1 Hz stimulation. Note that in the presence of the NO donor, facial stimulation failed to induce MLI-PC LTD in control mice.

Furthermore, we examined the effect of the NO donor on facial stimulation induced MLI-PC LTD in control mice. As shown in Figure 5; after the perfusion of SNAP (100 μM) for 1 h, delivery of 1 Hz facial stimulation failed to induce MLI-PC LTD in control mice (Figures 6A,B). The mean amplitude of P1 was 103.6 ± 8.1% of the baseline (100 ± 5.6%; Figure 6C; *P* = 0.67; *n* = 7) at 40–50 min after the trains of facial stimulation was delivered in control mice. The mean pause of SS firing was 104.4 ± 8.4% of the baseline (100 ± 7.9%; Figures 6A,D; *P* = 0.62) at 40–50 min after the trains of facial stimulation were delivered in the control mice. These results indicate that application of the NO donor prevents the MLI-PC LTD in control mice.

**Figure 7:**
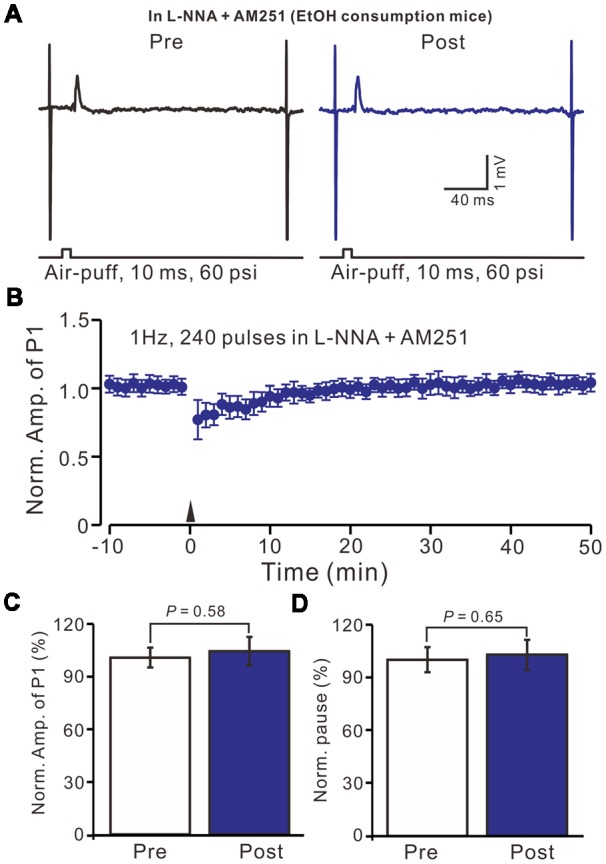
In the presence of the NO donor, SNAP (100 μM) and the CB1 receptor blocker, AM-251 (5 μM), facial stimulation induced MLI-PC LTP in control mice.** (A)** In the presence of SNAP and AM-251, representative traces showing air-puff stimulation (10 ms, 60 psi) evoked responses a cerebellar PC from a control mouse before and after delivering 1 Hz stimulation. **(B)** Summary of the normalized P1 amplitude after delivery of 1 Hz facial stimulation (arrow head) in the presence of SNAP and AM-251 in control mice (*n* = 6 mice). **(C)** Bar graph (*n* = 6 mice) showing the normalized amplitude of P1 before (Pre) and after (Post) delivery of 1 Hz stimulation. **(D)** Summary of data (*n* = 6 mice) showing the normalized pause of a simple spike firing before (Pre) and after (Post) delivery of 1 Hz stimulation. Note that in the presence of SNAP and AM-251, facial stimulation induced MLI-PC LTP in control mice.

We then observed the facial stimulation induced MLI-PC LTD, in the presence of the NO donor and the CB1 receptor blockers, in control mice. In the presence of SNAP and AM-251, 1 Hz facial stimulation induced MLI-PC LTP in control mice (Figures 7A,B). The mean amplitude of P1 was 114.6 ± 7.5% of the baseline (100 ± 8.2%; Figure 7C; *P* = 0.038; *n* = 6) at 40–50 min after 1 Hz facial stimulation. The mean pause of SS firing was 125.6 ± 9.3% of the baseline (100 ± 8.3%; Figures 7A,D; *P* = 0.024) at 40–50 min after 1 Hz facial stimulation in control mice. These results indicate that in the co-application of the NO donor and the CB1 receptor blockers, 1 Hz facial stimulation induces MLI-PC LTP rather than MLI-PC LTD, in control mice.

**Figure 8:**
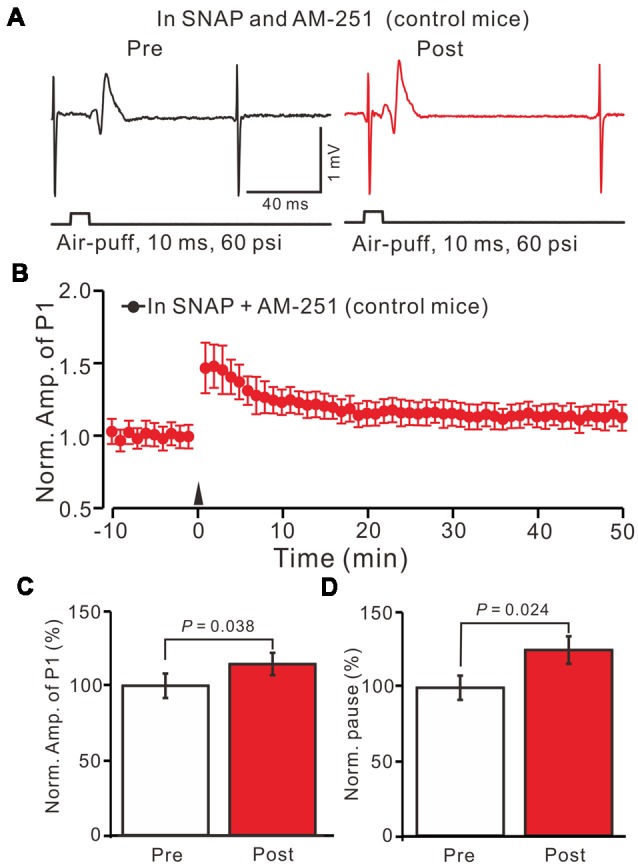
Inhibition of NOS and CB1 receptor activity, facial stimulation induced neither LTD nor LTP at MLI-PC synapses in EtOH consumption mice.** (A)** In the presence of the NOS inhibitor and the CB1 receptor blocker, representative traces showing air-puff stimulation (10 ms, 60 psi)-evoked responses in a cerebellar PC of EtOH consumption mice before (left) and after delivering 1 Hz stimulation. **(B)** Summary of the normalized P1 amplitude after delivery of 1 Hz facial stimulation (arrow head) in EtOH consumption mice (*n* = 7 mice). **(C)** Bar graph (*n* = 7 mice) showing the normalized amplitude of P1 before and after delivery of 1 Hz stimulation in EtOH consumption mice. **(D)** Summary of data (*n* = 7 mice) showing the normalized pause of a simple spike firing before and after delivery of 1 Hz stimulation in EtOH consumption. Note that at the inhibition of NOS and CB1 receptor activity, facial stimulation failed to induce MLI-PC LTD/LTP in EtOH consumption mice.

Moreover, we tested the facial stimulation induced MLI-PC LTD in the presence of the NOS inhibitor and the CB1 receptor blockers in EtOH consumption mice. In the presence of the NOS inhibitor and the CB1 receptor blockers, 1 Hz facial stimulation induced neither MLI-PC LTP nor MLI-PC LTD in EtOH consumption mice (Figures 8A,B). The mean amplitude of P1 was 102.3 ± 5.2% of the baseline (100 ± 7.3%; Figure 8C; *P* = 0.58; *n* = 7) at 40–50 min after the trains of 1 Hz facial stimulation were delivered in the EtOH consumption mice. The mean pause of SS firing was 103.1 ± 6.5% of the baseline (100 ± 7.6%; Figures 8A,D; *P* = 0.65) at 40–50 min after the facial stimulation were delivered in the EtOH consumption mice. These results indicate that inhibition of NOS and CB1 receptors activity, facial stimulation induces neither MLI-PC LTD nor MLI-PC LTP in EtOH consumption mice.

We also studied the effects of acute EtOH consumption on facial stimulation-induced MLI-PC synaptic plasticity. We found that acute EtOH consumption (1.6 g/kg) completely prevented MLI-PC LTD. However, by blocking CB1 receptor activity, neither MLI-PC LTD nor MLI-PC LTP was induced in acute EtOH consumption mice (not shown). These results indicated that acute EtOH consumption blocked MLI-PC LTD, was mainly related to the CB1 receptor *in vivo* in mice.

## Discussion

EtOH consumption causes alterations of motor coordination and learning, balance, behavior, speech, and certain cognitive functions which are considered to be caused partly by the impairment of the cerebellar circuit function. The main finding of this study is that facial stimulation induced MLI-PC LTD in control mice, but not in EtOH consumption mice. However, inhibition of NOS revealed a facial stimulation induced MLI-PC LTD, while blocking CB1 receptor activity uncovered a NO dependent MLI-PC LTP in EtOH consumption mice. Our results indicate that long-term EtOH consumption impairs the sensory stimulation-induced MLI–PC LTD *via* the activation of the NO signaling pathway in the cerebellar cortical Crus II *in vivo* in mice.

### EtOH Impairs the Facial Stimulation-Induced MLI-PC LTD *in vivo* in Mice

Cerebellar long-term synaptic plasticity has been proposed as a cellular mechanism for motor learning, which has been widely studied under *in vitro* conditions (Grasselli and Hansel, 2014). We previously found that facial stimulation at 1 Hz induced MLI-PC LTD *via* the activation of the NMDA receptor in a mouse cerebellar cortical Crus II, suggesting that the sensory stimulation evoked MLI-PC LTD, might contribute to motor learning in living animals (Bing et al., 2015). In this study, we showed that facial stimulation at 1 Hz induced MLI-PC LTD in control mice, but it failed to induce MLI-PC LTD in chronic EtOH consumption mice, suggesting that long-term consumption of EtOH impaired MLI-PC LTD *in vivo* in mice. The idea that EtOH impaired long-term synaptic plasticity in the central nervous system has been widely demonstrated (Izumi et al., 2005, 2007; Belmeguenai et al., 2008; Su et al., 2010; Tokuda et al., 2011; He et al., 2013; Zorumski et al., 2014). In the hippocampus CA1 region, acute administration of EtOH reversibly depresses LTD/LTP *via* modulating the NMDA and GABA_A_ receptors (Izumi et al., 2005), as well as the GABAergic neurosteroids (Izumi et al., 2007; Tokuda et al., 2011). In the cerebellum, acute application of EtOH inhibits LTD at either CF-PC or PF-PC synapses *via* inhibition of the NMDA receptors and the group I mGlu receptors (Carta et al., 2006; Belmeguenai et al., 2008; Su et al., 2010; He et al., 2013).

Chronic application of EtOH impairing memory and synaptic plasticity, has also been demonstrated by previous studies (Van Waes et al., 2009; Mishra et al., 2012; Wills et al., 2017). Chronic EtOH exposure reduces performance in spatial recognition tasks in normal animals, but it attenuates spatial memory deficits and increases the mGlu1 receptor expression in the hippocampus of prenatal stress rats (Van Waes et al., 2009). Further, chronic intermittent exposure of EtOH disrupts the NMDA receptor-associated post-synaptic proteins and specifically regulates the group I mGlu receptor-dependent LTD in the mouse hippocampus (Wills et al., 2017). In addition, chronic EtOH consumption significantly reduces simple and complex spike frequencies of PCs, resulting in a depression of cerebellar motor coordination and ataxia in mice (Servais et al., 2005). Our present study is consistent with previous studies, indicating that chronic exposure to EtOH impairs facial stimulation-induced MLI-PC LTD, suggesting that long-term consumption of EtOH may cause a deficit in the cerebellar motor learning function *in vivo* in mice.

### CB1 Receptors Play a Critical Role in the Facial Stimulation-Evoked MLI-PC Long-Term Synaptic Plasticity *in vivo* in Mice

In the cerebellar cortex, eCB is generated and released from cerebellar PCs and MLIs during the tetanus stimulation (Brown et al., 2003; Beierlein and Regehr, 2006; Soler-Llavina and Sabatini, 2006). We previously found that CB1 receptor-dependent PF–PC presynaptic LTD was observed *in vivo* in the absence of a pharmacological blocker, suggesting that eCB signaling under *in vivo* conditions is stronger than that under *in vitro* conditions (Chu et al., 2014). In addition, CB 1 receptor-dependent presynaptic LTD was induced when presynaptic LTP was pharmacologically blocked in mouse cerebellar slices, suggesting that the eCB signaling pathway plays a critical role in cerebellar cortical neuronal plasticity (Qiu and Knöpfel, 2009) under control conditions. We recently found that blocking the CB1 receptors abolished facial stimulation evoked MLI–PC LTD, indicating that sensory stimulation induced MLI-PC LTD, *via* the eCB signaling pathway (Bing et al., 2015). Our present results showed that by blocking CB1 receptor activity, facial stimulation failed to induce MLI-PC LTD in control mice, but the stimulation induced a MLI-PC LTP in EtOH consumption mice, indicating that chronic EtOH consumption impaired MLI-PC LTD.

### Chronic EtOH Exposure Impairs Facial Stimulation-Induced MLI-PC LTD *via* Enhancement of the NO Signaling Pathway

It is known that NO can either facilitate or suppress plasticity through biphasic effects on NMDA receptors and AMPA receptors insertion, presynaptic vesicle regulation, S-nitrosylation of synaptic proteins, and cyclic GMP (cGMP) generation (Reyes-Harde et al., 1999; Stanton et al., 2005; Ratnayaka et al., 2012; Selvakumar et al., 2013). In the cerebellar cortex, NO works as a retrograde signal, and has been implicated in presynaptically expressed LTP (Qiu and Knöpfel, 2007; Chu et al., 2014). In this study, facial stimulation induced MLI-PC LTP in the absence of CB1 receptor activity, which was blocked by the NOS inhibitor. However, facial stimulation induced MLI-PC LTD, in EtOH consumption mice in the absence of NOS activity. These results indicate that chronic EtOH consumption impairs MLI-PC LTD, may relate to the enhancement of NOS activity *in vivo* in mice Moreover, the application of the NO donor prevented the facial stimulation induced MLI-PC LTD in control mice, suggesting that an increase of NO levels inhibits the induction of MLI-PC LTD. Our results are supported by several previous studies (Yu et al., 2013; Finnerty et al., 2015; Lo et al., 2018), suggesting that EtOH consumption increases the NO level under *in vivo* conditions. First, systemic administration of EtOH resulted in a dose-dependent increase in NO levels, which was attenuated by administration of the NOS inhibitor (Finnerty et al., 2015). Furthermore, acute treatment with EtOH increased the NOS activity and NO production in the brain regions associated with memory, including the prefrontal cortex, amygdala and the hippocampus of adult mice (Yu et al., 2013). Moreover, EtOH consumption causes an increase in the level of glutamate, NO, and GABA in the rostral ventrolateral medulla during the hypotensive responses, suggesting that EtOH enhances the glutamatergic NMDA receptor/NO/GABA pathways in the rostral ventrolateral medulla and may participate in the hypotensive effects induced by acute administration of EtOH (Lo et al., 2018).

Altogether, our present study demonstrates that long-term EtOH consumption can impair the sensory stimulation-induced LTD at MLI–PC synapses, *via* the activation of the NO signaling pathway in the cerebellar cortical Crus II *in vivo* in mice, suggesting that the EtOH consumption attenuates motor coordination and motor learning, which may be involved in the impairment of MLI–PC synaptic plasticity *in vivo* in mice.

## Author Contributions

D-LQ, XC, L-DS conceived and designed the experiments. D-YL, Y-HB performed the experiments. C-PC, DL-Q analyzed the data. Y-HB contributed reagents, materials and analysis tools. C-PC, S-BC, D-LQ, L-DS wrote the manuscript.

## Conflict of Interest Statement

The authors declare that the research was conducted in the absence of any commercial or financial relationships that could be construed as a potential conflict of interest.

## References

[B1] Anggono V. Huganir R. L. (2012). Regulation of AMPA receptor trafficking and synaptic plasticity. Curr. Opin. Neurobiol. 22, 461–469. 10.1016/j.conb.2011.12.006 22217700 PMC3392447

[B2] Beierlein M. Regehr W. G. (2006). Local interneurons regulate synaptic strength by retrograde release of endocannabinoids. J. Neurosci. 26, 9935–9943. 10.1523/JNEUROSCI.0958-06.2006 17005857 PMC6674464

[B3] Belmeguenai A. Botta P. Weber J. T. Carta M. De Ruiter M. De Zeeuw C. I.. (2008). Alcohol impairs long-term depression at the cerebellar parallel fiber-Purkinje cell synapse. J. Neurophysiol. 100, 3167–3174. 10.1152/jn.90384.2008 18922952 PMC2604851

[B4] Bing Y. H. Wu M. C. Chu C. P. Qiu D. L. (2015). Facial stimulation induces long-term depression at cerebellar molecular layer interneuron-Purkinje cell synapses *in vivo* in mice. Front. Cell. Neurosci. 9:214. 10.3389/fncel.2015.00214 26106296 PMC4460530

[B5] Botta P. de Souza F. M. Sangrey T. De Schutter E. Valenzuela C. F. (2010). Alcohol excites cerebellar Golgi cells by inhibiting the Na^+^/K^+^ ATPase. Neuropsychopharmacology 35, 1984–1996. 10.1038/npp.2010.76 20520600 PMC2904864

[B6] Brown S. P. Brenowitz S. D. Regehr W. G. (2003). Brief presynaptic bursts evoke synapse-specific retrograde inhibition mediated by endogenous cannabinoids. Nat. Neurosci. 6, 1048–1057. 10.1038/nn1126 14502290

[B7] Carta M. Mameli M. Valenzuela C. F. (2006). Alcohol potently modulates climbing fiber-Purkinje neuron synapses: role of metabotropic glutamate receptors. J. Neurosci. 26, 1906–1912. 10.1523/JNEUROSCI.4430-05.2006 16481422 PMC6674936

[B8] Chandler L. J. Harris R. A. Crews F. T. (1998). Ethanol tolerance and synaptic plasticity. Trends Pharmacol. Sci. 19, 491–495. 10.1016/s0165-6147(98)01268-1 9871410

[B11] Chu C. P. Bing Y. H. Liu Q. R. Qiu D. L. (2011a). Synaptic responses evoked by tactile stimuli in Purkinje cells in mouse cerebellar cortex. PLoS One 6:e22752. 10.1371/journal.pone.0022752 21818384 PMC3144243

[B9] Chu C. P. Bing Y. H. Qiu D. L. (2011b). Sensory stimulation evokes inhibition rather than excitation in cerebellar PCs *in vivo* in mice. Neurosci. Lett. 487, 182–186. 10.1016/j.neulet.2010.10.018 20965231

[B10] Chu C. P. Bing Y. H. Liu H. Qiu D. L. (2012). Roles of molecular layer interneurons in sensory information processing in mouse cerebellar cortex Crus II *in vivo*. PLoS One 7:e37031. 10.1371/journal.pone.0037031 22623975 PMC3356402

[B12] Chu C. P. Zhao G. Y. Jin R. Zhao S. N. Sun L. Qiu D. L. (2014). Properties of 4 Hz stimulation-induced parallel fiber-Purkinje cell presynaptic long-term plasticity in mouse cerebellar cortex *in vivo*. Eur. J. Neurosci. 39, 1624–1631. 10.1111/ejn.12559 24666426

[B13] Criswell H. E. Breese G. R. (2005). A conceptualization of integrated actions of ethanol contributing to its GABAmimetic profile: a commentary. Neuropsychopharmacology 30, 1407–1425. 10.1038/sj.npp.1300750 15856077

[B14] Cui S. B. Cui B. R. Liu H. Wu M. C. Xu Y. H. Bian J. H.. (2014). Effects of ethanol on sensory stimulus-evoked responses in the cerebellar molecular layer *in vivo* in mice. Neurosci. Lett. 577, 112–116. 10.1016/j.neulet.2014.05.037 24861511

[B15] D’Angelo E. Mapelli L. Casellato C. Garrido J. A. Luque N. Monaco J.. (2016). Distributed circuit plasticity: new clues for the cerebellar mechanisms of learning. Cerebellum 15, 139–151. 10.1007/s12311-015-0711-7 26304953

[B16] Finnerty N. O’Riordan S. L. Klamer D. Lowry J. Pålsson E. (2015). Increased brain nitric oxide levels following ethanol administration. Nitric Oxide 47, 52–57. 10.1016/j.niox.2015.03.002 25819134

[B17] Grasselli G. Hansel C. (2014). Cerebellar long-term potentiation: cellular mechanisms and role in learning. Int. Rev. Neurobiol. 117, 39–51. 10.1016/B978-0-12-420247-4.00003-8 25172628

[B19] He Q. Titley H. Grasselli G. Piochon C. Hansel C. (2013). Ethanol affects NMDA receptor signaling at climbing fiber-Purkinje cell synapses in mice and impairs cerebellar LTD. J. Neurophysiol. 109, 1333–1342. 10.1152/jn.00350.2012 23221414 PMC3602830

[B21] Hirano T. (2013). “GABA and synaptic transmission in the cerebellum,” in Handbook of the Cerebellum and Cerebellar Disorders, eds Manto M. Schmahmann J. D. Rossi F. Gruol D. L. Koibuchi N. (Heidelberg: Springer), 881–893.

[B20] Hirano T. Kawaguchi S. Y. (2014). Regulation and functional roles of rebound potentiation at cerebellar stellate cell-Purkinje cell synapses. Front. Cell. Neurosci. 8:42. 10.3389/fncel.2014.00042 24600347 PMC3927423

[B22] Hirono M. Yamada M. Obata K. (2009). Ethanol enhances both action potential-dependent and action potential-independent GABAergic transmission onto cerebellar Purkinje cells. Neuropharmacology 57, 109–120. 10.1016/j.neuropharm.2009.04.012 19426745

[B23] Hoxha E. Tempia F. Lippiello P. Miniaci M. C. (2016). Modulation, plasticity and pathophysiology of the parallel fiber-purkinje cell synapse. Front. Synaptic Neurosci. 8:35. 10.3389/fnsyn.2016.00035 27857688 PMC5093118

[B24] Ito M. (1989). Long-term depression. Annu. Rev. Neurosci. 12, 85–102. 10.1146/annurev.ne.12.030189.000505 2648961

[B25] Izumi Y. Murayama K. Tokuda K. Krishnan K. Covey D. F. Zorumski C. F. (2007). GABAergic neurosteroids mediate the effects of ethanol on long-term potentiation in rat hippocampal slices. Eur. J. Neurosci. 26, 1881–1888. 10.1111/j.1460-9568.2007.05809.x 17883414

[B26] Izumi Y. Nagashima K. Murayama K. Zorumski C. F. (2005). Acute effects of ethanol on hippocampal long-term potentiation and long-term depression are mediated by different mechanisms. Neuroscience 136, 509–517. 10.1016/j.neuroscience.2005.08.002 16216426

[B27] Lo H. Lin H. H. Chen J. K. Situmorang J. H. Lai C. C. (2018). Involvement of NMDA receptors, nitric oxide and GABA in rostral ventrolateral medulla in acute ethanol-induced cardiovascular responses in rats. Alcohol. Clin. Exp. Res. [Epub ahead of print]. 10.1111/acer.13800 29846938

[B28] Lovinger D. M. (1997). Alcohols and neurotransmitter gated ion channels: past, present and furture. Naunyn Schmiedebergs Arch. Pharmacol. 356, 267–282. 10.1007/pl00005051 9303562

[B29] Luo J. (2012). Mechanisms of ethanol-induced death of cerebellar granule cells. Cerebellum 11, 145–154. 10.1007/s12311-010-0219-0 20927663 PMC3355343

[B30] Mameli M. Botta P. Zamudio P. A. Zucca S. Valenzuela C. F. (2008). Ethanol decreases Purkinje neuron excitability by increasing GABA release in rat cerebellar slices. J. Pharmacol. Exp. Ther. 327, 910–917. 10.1124/jpet.108.144865 18755936 PMC2768120

[B31] Martz A. Deitrich R. A. Harris R. A. (1983). Behavioral evidence for the involvement of γ-aminobutyric acid in the actions of ethanol. Eur. J. Pharmacol. 89, 53–62. 10.1016/0014-2999(83)90607-6 6861890

[B32] Mishra D. Zhang X. Chergui K. (2012). Ethanol disrupts the mechanisms of induction of long-term potentiation in the mouse nucleus accumbens. Alcohol. Clin. Exp. Res. 36, 2117–2125. 10.1111/j.1530-0277.2012.01824.x 22551245

[B33] Palay S. L. Chan-Palay V. (1974). Cerebellar Cortex. New York, NY: Springer-Verlag.

[B34] Qiu D. L. Knöpfel T. (2007). An NMDA receptor/nitric oxide cascade in presynaptic parallel fiber-Purkinje neuron long-term potentiation. J. Neurosci. 27, 3408–3415. 10.1523/JNEUROSCI.4831-06.2007 17392457 PMC6672131

[B35] Qiu D. L. Knöpfel T. (2009). Presynaptically expressed long-term depression at cerebellar parallel fiber synapses. Pflugers Arch. 457, 865–875. 10.1007/s00424-008-0555-9 18663469

[B36] Ratnayaka A. Marra V. Bush D. Burden J. J. Branco T. Staras K. (2012). Recruitment of resting vesicles into recycling pools supports NMDA receptor-dependent synaptic potentiation in cultured hippocampal neurons. J. Physiol. 590, 1585–1597. 10.1113/jphysiol.2011.226688 22271866 PMC3413500

[B37] Reyes-Harde M. Empson R. Potter B. V. Galione A. Stanton P. K. (1999). Evidence of a role for cyclic ADP-ribose in long-term synaptic depression in hippocampus. Proc. Natl. Acad. Sci. U S A 96, 4061–4066. 10.1073/pnas.96.7.4061 10097163 PMC22420

[B38] Schmahman J. D. Sherman J. C. (1997). Cerebellar cognitive affective syndrome. Int. Rev. Neurobiol. 41, 433–440. 10.1016/S0074-7742(08)60363-3 9378601

[B39] Selvakumar B. Jenkins M. A. Hussain N. K. Huganir R. L. Traynelis S. F. Snyder S. H. (2013). S-nitrosylation of AMPA receptor GluA1 regulates phosphorylation, single-channel conductance and endocytosis. Proc. Natl. Acad. Sci. U S A 110, 1077–1082. 10.1073/pnas.1221295110 23277581 PMC3549090

[B40] Servais L. Bearzatto B. Delvaux V. Noël E. Leach R. Brasseur M.. (2005). Effect of chronic ethanol ingestion on Purkinje and Golgi cell firing *in vivo* and on motor coordination in mice. Brain Res. 1055, 171–179. 10.1016/j.brainres.2005.07.026 16107247

[B41] Soler-Llavina G. J. Sabatini B. L. (2006). Synapse-specific plasticity and compartment- alized signaling in cerebellar stellate cells. Nat. Neurosci. 9, 798–806. 10.1038/nn1698 16680164

[B42] Stanton P. K. Winterer J. Zhang X. L. Müller W. (2005). Imaging LTP of presynaptic release of FM1–43 from the rapidly recycling vesicle pool of Schaffer collateral-CA1 synapses in rat hippocampal slices. Eur. J. Neurosci. 22, 2451–2461. 10.1111/j.1460-9568.2005.04437.x 16307588

[B43] Su L. D. Sun C. L. Shen Y. (2010). Ethanol acutely modulates mGluR1-dependent long-term depression in cerebellum. Alcohol. Clin. Exp. Res. 34, 1140–1145. 10.1111/j.1530-0277.2010.01190.x 20477778

[B44] Tokuda K. Izumi Y. Zorumski C. F. (2011). Ethanol enhances neurosteroidogenesis in hippocampal pyramidal neurons by paradoxical NMDA receptor activation. J. Neurosci. 31, 9905–9909. 10.1523/JNEUROSCI.1660-11.2011 21734282 PMC3180997

[B45] Van Waes V. Enache M. Zuena A. Mairesse J. Nicoletti F. Vinner E.. (2009). Ethanol attenuates spatial memory deficits and increases mGlu1a receptor expression in the hippocampus of rats exposed to prenatal stress. Alcohol. Clin. Exp. Res. 33, 1346–1354. 10.1111/j.1530-0277.2009.00964.x 19413649

[B46] Wadleigh A. Valenzuela C. F. (2012). Ethanol increases GABAergic transmission and excitability in cerebellar molecular layer interneurons from GAD67-GFP knock-in mice. Alcohol Alcohol. 47, 1–8. 10.1093/alcalc/agr147 22080831 PMC3243440

[B47] Weiner J. L. Valenzuela C. F. (2006). Ethanol modulation of GABAergic transmission: the view from the slice. Pharmacol. Ther. 111, 533–554. 10.1016/j.pharmthera.2005.11.002 16427127

[B48] Wills T. A. Baucum A. J. Holleran K. M. Chen Y. Pasek J. G. Delpire E.. (2017). Chronic intermittent alcohol disrupts the GluN2B-associated proteome and specifically regulates group I mGlu receptor-dependent long-term depression. Addict Biol. 22, 275–290. 10.1111/adb.12319 26549202 PMC4860359

[B49] Woodward J. J. (1999). Ionotropic glutamate receptors as sites of action for ethanol in the brain. Neurochem. Int. 35, 107–113. 10.1016/s0197-0186(99)00052-2 10405994

[B50] Wu M. C. Bing Y. H. Chu C. P. Qiu D. L. (2016). Ethanol modulates facial stimulation-evoked outward currents in cerebellar Purkinje cells *in vivo* in mice. Sci. Rep. 6:30857. 10.1038/srep30857 27489024 PMC4973232

[B51] Yu S. Y. Gao R. Zhang L. Luo J. Jiang H. Wang S. (2013). Curcumin ameliorates ethanol-induced memory deficits and enhanced brain nitric oxide synthase activity in mice. Prog. Neuropsychopharmacol. Biol. Psychiatry 44, 210–216. 10.1016/j.pnpbp.2013.03.001 23500667

[B52] Zorumski C. F. Mennerick S. Izumi Y. (2014). Acute and chronic effects of ethanol on learning-related synaptic plasticity. Alcohol 48, 1–17. 10.1016/j.alcohol.2013.09.045 24447472 PMC3923188

